# An *In Vitro* Evaluation of Antioxidant and Colonic Microbial Profile Levels following Mushroom Consumption

**DOI:** 10.1155/2013/289821

**Published:** 2013-08-20

**Authors:** Emanuel Vamanu, Diana Pelinescu, Ionela Avram, Sultana Nita

**Affiliations:** ^1^Department of Industrial Biotechnology, University of Agronomical Sciences and Veterinary Medicine Bucharest, Faculty of Biotechnology, 59 Marasti Boulevard, 011464 Bucharest, Romania; ^2^University of Bucharest, Faculty of Biology, 1-3 Portocalilor Street, 5 District, 060101 Bucharest, Romania; ^3^National Institute of Chemical-Pharmaceutical Research and Development (ICCF), 112 Vitan Road, Bucharest, Romania

## Abstract

The biological activity of mushroom consumption is achieved by the antioxidant effect of constituent biomolecules released during digestion. In the following study, the consumption of mushroom fungi was determined to increase the number of *Lactobacillus* and *Bifidobacterium* strains within the colon. The main phenolic antioxidant compounds identified were both gentisic and homogentisic acids. Moreover, the flavonoid catechin as well as a significant amount of **δ**- and **γ**-tocopherols was determined. The amount of *Lactobacillus* and *Bifidobacterium* strains from different sections of the human colon was significantly correlated with levels of antioxidative biomolecules. The experimental data clearly demonstrate a significant impact of mushroom consumption on the fermentative function of microorganisms in the human colon, resulting in the homeostasis of normal physiological colonic functions.

## 1. Introduction

The colon microflora is continuously influenced by food intake. The presence of certain nutrients and the metabolic activity of microorganisms that colonize the colon regions both play an significant role in the well-being of individuals. The ability to modulate immune functions by probiotic bacteria and the ability to metabolize carcinogen agents as well as the buffering capacity exerted against the pathogenic strains are amongst several of many roles that the colon microflora performs. 

European researchers have only recently begun to further investigate the healing or medicinal capabilities of edible mushrooms [1]. In addition to the bioactive compounds with pharmaceutical efficacy (phenolic compounds), mushrooms are known to contain significant amounts of proteins and dietary fibers. It appears as though no direct linkage between the ingested amounts of antioxidant phenols and the assimilation of these phenols within the body has been found to date. It is therefore important to determine whether these phenolic compounds are in fact degraded by microbial metabolism at the colon level. Due to the high fiber content of mushrooms, they represent a good natural food source for maintaining the human colonic microflora in its appropriate equilibrium status [2].

Currently, the most efficient and reproducible method for investigating the effect of a dietary supplement or food on human colonic microflora and of the effect manifested by its composition is the use of *in vitro* simulation systems. *In vitro* simulation systems have gained attention compared to *in vivo* systems due to the *in vivo* systems ethical limitations, increased time consumption, and increased cost. The aim of our study was to investigate the effects of the intake of four edible mushrooms on the fermentative capacity of the simulated microbiota of the colon. Simulation of the three regions of the colon was achieved by using the GIS1 unicameral system. During the transverse passage through the colon segments, the differential antioxidant activity following consumption of the various mushroom types (*Boletus edulis, Pleurotus ostreatus, Agaricus bisporus, *and* Cantharellus cibarius*) was evaluated. The antioxidative efficacy was then correlated with the amount of phenols, flavonoids, and favorable microorganisms (*Lactobacillus *and* Bifidobacterium strains*).

## 2. Materials and Methods

*Pleurotus ostreatus, A. bisporus, *and* C. cibarius *mushrooms were purchased from supermarkets in Bucharest, and *B. edulis* mushrooms were harvested from the forests of Gorj County, Romania. Selected fungi mushrooms were washed, and all chemicals and reagents were purchased from Sigma-Aldrich (St. Louis, MO, USA). All other unlabeled chemicals and reagents were of analytical grade.

### 2.1. Determination of Total Antioxidant Activity

The antioxidant activity of extracts was measured by determining the 1,1-diphenyl-2-picrylhydrazyl (DPPH) free radical scavenging capacity. Samples (100 *μ*L) were mixed with 3 mL of ethanol solution of 0.004% of DPPH, and the absorbance was read at 517 nm after 30 min, with a Helios *λ* spectrophotometer [3]. The antioxidant activity (DPPH radical scavenging activity) was calculated using the following equation (%) of antioxidant activity: 1 − (As/Ac) × 100, where As is the absorbance in the presence of sample and Ac is the absorbance in the absence of sample.

### 2.2. Measurement of Reducing Power

Each sample was mixed with 2.5 mL of 200 mM sodium phosphate buffer (pH 6.6) and 2.5 mL of 1% potassium ferricyanide, and the mixture was incubated at 50°C for 20 min. Next, 2.5 mL of 10% trichloroacetic acid was added, and the mixture was centrifuged at 3000 ×g for 10 min. The upper layer (2.5 mL) was mixed with 2.5 mL of deionized water and 0.5 mL of 0.1% ferric chloride. Finally, the absorbance was measured at 700 nm [4, 5].

### 2.3. Determining the Compounds with Antioxidant Effects

The determination of polyphenol carboxylic acids, flavones, and tocopherols was performed by HPLC (ELITE-LaChrom, with DAD detector) and was presented in a previous study [5].

Total soluble phenolic content was estimated by the Folin-Ciocalteau method, and the total soluble flavonoid content was estimated by an aluminium chloride colorimetric method [6, 7].

### 2.4. *In Vitro* Gastrointestinal Simulation

#### 2.4.1. *In Vitro *Digestion Protocol

A 500 mL Duran bottle provided with a stopper having three entries for additions was used. The digestion process was performed individually for each used fungus at a temperature of 37°C using a heated plate with a temperature sensor. Twenty grams of dried mushrooms were mixed with 10 mL physiological serum, and a simulated gastric phase formulated using 3 mL pepsin (40 mg/mL) and pH corrected to 2 with HCl 0.1 N was added. The mixture was maintained at the specified pH for approximately 2 h with slight continuous shaking. For the small intestinal phase, the pH was adjusted to 5.4 with 1 M Na_2_CO_3_, and 9 mL pancreatin (2 mg/mL) and bile salts (3 mg/mL) were subsequently added, maintaining the mixture under the above-mentioned conditions for an additional 2 h. Finally, the parts of digested fungus were then stored for subsequent introduction to the colon simulation system GIS1—phase 2 [8].

#### 2.4.2. *In Vitro *Human Colon Simulation

Conditions in the colon were replicated in a single-chamber system, GIS1, inoculated with 10% (wt:v) fecal homogenate from a child (4 years old) in peptone water and with 1% (wt:v) mushrooms introduced to the digestion process. After inoculation, the GIS1 was left for approximately 24 h for the stabilization period. The system was operated for 20 h [9]. The GIS1 system was described in a previous study [10]. The control represents only microflora simulation.

Determination of dry matter disappearance was performed according to the method described by Goñi et al., 2005 [11]. 

For determination of the total quantity of dietary fibers, the AOAC 985.29 method was used [12]. 

### 2.5. Microbiological Analysis

Analyses were performed by serially diluting the culture sample in physiological saline solution (pH 7.0). The two highest dilutions were subsequently plated on specific media and evaluated by an automated colony counter, ColonyQuant, with the corresponding software [13, 14]. 

A total number of anaerobes and facultative anaerobes were determined by using anaerobe agar and nutrient agar, Mac Conkey agar for coliforms, Azide blood agar base for enterococci, mannitol salt agar for staphylococci, sulfite polymyxin sulfadiazine agar for clostridia, Rogosa agar for lactobacilli, and Beerens' medium agar for bifidobacteria. Media were purchased from Oxoid (Hampshire, UK).

### 2.6. Relative Quantification of Bacteria Number

For the quantitation of the number of total microorganisms and of those belonging to the genera *Lactobacillus* and *Bifidobacterium*, reverse transcriptase- (RT-) PCR was applied using three pairs of primers (Table 1). A pair of universal primers for prokaryotes (total number of microorganisms) was used, and pairs of primers that allow amplification of specific sequences from the chromosomal DNA of bacteria of the genera *Lactobacillus* and *Bifidobacterium*. In the phase of gene quantification, the kit SYBR Green RT-PCR Reagents (Applied Biosystems, Foster City, CA, USA) was used. In the amplification reactions 50 ng DNA and 0.2 *μ*M primer were included, in a final volume of 20 *μ*L. The samples were run using the system 7900 fast real-time PCR (Applied Biosystems) according to the following program: step 1, 95°C, 10 min; step 2, denaturation 95°C, 15 s; step 3, alignment/elongation, 60°C 1 min (steps 2-3 further repeated for additional 40 times). Following amplification, the results were interpreted using RQ Manager software (Applied Biosystems), and the threshold value with determined Ct was 0.0884 [15, 16].

### 2.7. Metabolic Activity Analysis

Ammonia quantification from the samples was evaluated by use of an ammonia assay kit, catalog no. AA0100 from Sigma-Aldrich [17].

Lactate concentrations were measured by BioAssay Systems' EnzyChrom lactate assay kit [18].

### 2.8. Statistical Analysis

All parameters for antimicrobial and antioxidant activities were assessed in triplicate, and the results were expressed as mean ± SD values. The mean values and standard deviations were calculated using the EXCEL program from Microsoft Office 2010 package. Statistical analysis was carried out using GraphPad Prism 6.0. Significance level was set at *P* ≤ 0.05.

## 3. Results and Discussions

### 3.1. *In Vitro* Antioxidant Activity following Mushroom Consumption

**Figure 1:**
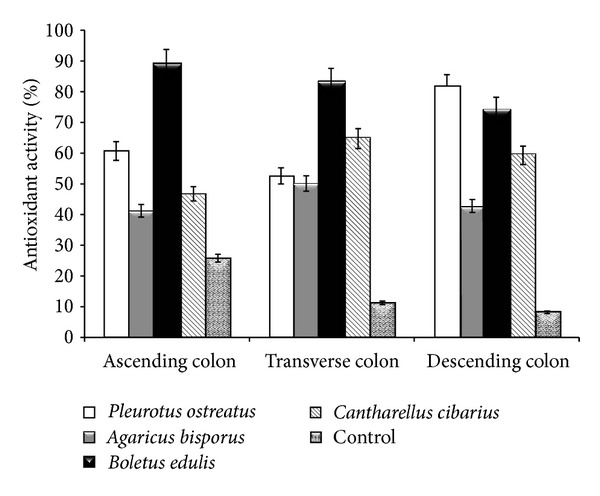
Antioxidant activity in the colon regions following mushroom consumption.

Typically, studies regarding the antioxidant action of various functional foods are limited to the digestion performed at the stomach and at the small intestinal levels. The evaluation of antioxidant activity by means of DPPH scavenging ability is an accurate assessment due to DPPH radical stability in comparison to the complexity of physiological processes and to the microbial variety in the human colon [19]. The determined values demonstrated a varied antioxidant activity, which differs from one colon segment to another (Figure 1). In the ascending region, the antioxidant activity ranged 41.00–88.68% and 50.00–83.09% in the transverse region and 42.33–81.47% in the descending region of the colon.

Consumption of *A. bisporus* caused the lowest antioxidant activity in each simulated region. Overall, the consumption of the *B. edulis* mushrooms demonstrated the highest antioxidant potential in the first two regions, with the exception of the descending region, where the consumption of *P. ostreatus* exceeded the determined value by approximately 9.5%. The data is in agreement with previous studies aimed at *in vitro* biochemical determinations, which showed significant antioxidant capacity of both *B. edulis* and *C. cibarius* extracts. In contrast, *A. bisporus* is known to have a much lower antioxidative capacity in comparison to most wild edible mushrooms [20].

Except for *P. ostreatus* and *C. cibarius*, the trend is to decrease the antioxidant capacity determined by DPPH scavenging activity analysis, with the mushroom passage from one compartment to another in the colon. Overall, the maximum antioxidant capacity was determined in the transverse segment, a process due to the fermentative action of microflora, leading to the release of biomolecules with antioxidant efficacy. According to previous studies, biomolecules are associated with the insoluble fraction represented by a unique group of polyphenols (proanthocyanidins), which can be fermented by the simulated microflora of the colon [21]. This insoluble fraction which resisted the attack caused by low gastric pH and by bile has a maximum antioxidant effect in the transverse segment, being released within 6 to 8 h. Although it was considered to be without biological activity because it was released in the feces, several studies have demonstrated its degradation by the human colonic microflora into low-molecular-weight aromatic acids [22]. While its discharge into the simulated system can be observed by increasing the antioxidant activity, the complete degradation takes up to 48 h [23]. With the exception of *P. ostreatus*, antioxidant activity is due to the composition and to a more difficult digestion, which makes the release of biomolecules occur within a longer time span. This time interval with increased antioxidant activity represented a characteristic property of the tested fungi. This time period corresponded with the complete digestion of the amount of tested mushroom (ingested).

### 3.2. Reducing Power

**Figure 2:**
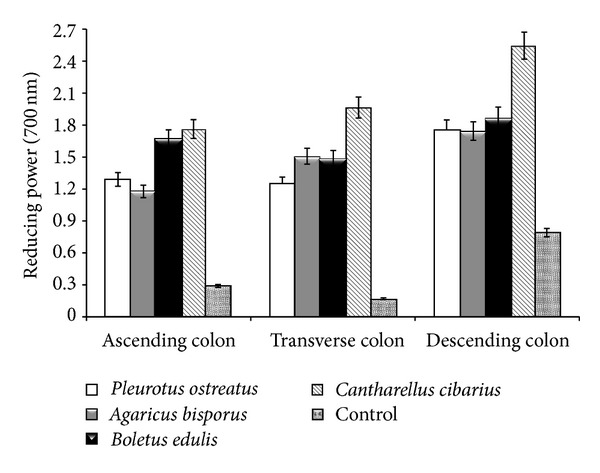
Reducing power in the colon regions following mushroom consumption.

The reducing power is a direct indicator of antioxidant capacity. According to the present study data (Figure 2), a significant amount of reductones was released by the digestion process. Reductones through interaction with free radicals are shown to block the free radicals ability to react [24]. The results however differ significantly depending on the regions of the colon and on the mushroom type consumed. *C. cibarius* and *B. edulis* showed the highest reducing power. *C. cibarius* showed an increase in reduction power with its passage from one region to another, while the consumption of the *B. edulis* mushrooms caused a relatively constant reduction power that varied on average within ±15%. The use of the *in vitro* GIS1 system helped determine the antioxidant behavior of mushroom consumption. Wild edible mushrooms (*B. edulis* and *C. cibarius*) caused a maximal increase of reduction power by approximately 30% in the terminal region of the human colon. 

The fermentative action of microflora caused an increase in the reduction power following 7 h of direct contact with the transverse region and continued with maximal values in the descending colon region. *P. ostreatus* consumption was exceptional in not causing such behavior, but, for the rest of the fungi types, the increase of the values ranged 25–30%. This characteristic behavior, which in the case of mushroom consumption is correlated to antioxidant activity. As for the segmental region of the colon and the fermentation time period, there is no data in the literature that can explain this phenomenon. 

The fermentative action process was expressed more for *B. edulis*, which has completely disintegrated halfway through the stationary phase of the transverse colonic region. In contrast, for both *P. ostreatus* and *A. bisporus*, the same process was slow. In the final stage of digestion, remaining mushrooms were not completely disintegrated, in particular with *A. bisporus* consumption. 

### 3.3. Microbiological Analysis

Due to the impact of the colonic microflora on the immune system as well as its role in the digestive process, the analysis of colonic microflora may therefore be considered as an indirect indicator of the quality of consumed food [25]. The increased acknowledgment of the beneficial health implications of various biomolecules with antioxidant properties warranted a study of their effects on the colon microflora. The effect that antioxidative biomolecules have on the various microbial strains and the correlation between the antioxidant status and the number of *Lactobacillus* and *Bifidobacterium* strains further validate the significant role that ingested food has on human health. By testing resistance it was shown that the species of *Lactobacillus* and *Bifidobacterium* genera showed resistance to extracts from *Lentinula edodes*. However, most genera containing pathogenic species were inhibited at low concentrations of the tested solutions [26].

Thus, the use of selective media to determine the relationship between the groups of microorganisms which constitute the colonic microflora showed that the consumption of mushrooms has a significant influence. *C. cibarius* and *P. ostreatus* caused a gradual decrease in the number of staphylococci, with the transition from one segment to another. This demonstrates that the release of bioactive molecules with antimicrobial effect is mainly due to a fermentative action of the microflora. The observed differences reached up to approximately 1 log CFU/mL in the descending segment of the colon. For clostridia and coliforms, a limited increase of CFU value was noted which did not exceed 0.5 log CFU/mL in the ascending segment. Conversely, in the transverse and descending segments of the colon, a limited reduction in the number of these microbial groups was noted (Table 1).

The most significant effect as a result of the consumption of the tested fungi was, in this situation, the increase in the number of *Lactobacillus* and *Bifidobacterium* strains. The number of lactobacilli was greatest in the transverse and descending colonic segments. The maximum value determined for *B. edulis* was approximately 1.5 log CFU/mL higher than the control with the exception of the consumption of *B. edulis*, which resulted in a limited increase of lactobacilli number possibly due to the antimicrobial effect. Following the digestion process a number of compounds are released and known to affect the viability of these bacteria, an effect that was also determined for *C. cibarius*. This observation was not noted for the number of bifidobacteria, as the cell number registered an overall decrease. In the case of the consumption of the two wild edible mushroom types, an average reduction in the number of bifidobacteria was recorded, ranging 1.28–1.48 log CFU/mL.

For the respective measurements in both the quantitative and qualitative changes of microorganisms in analyzed samples, the total genomic DNA was extracted. The spectrophotometric electrophoretic analysis revealed that there are variations in terms of concentration and purity of the extracted DNA. These variations that have occurred are due both to differences in the microflora of each region of the colon and to the direct influence of the consumed fungus. The analysis was focused primarily on the *Lactobacillus* and *Bifidobacterium* strains. The extracts of genomic DNA from the analyzed samples were used in the experiments of quantification of the relative number of microorganisms, by applying real-time PCR technique. To quantify the number of microorganisms, the average of Ct values (threshold cycle) was determined (Table 2).

Ct values were inversely proportional to the number of copies of analyzed genes. Thus a low Ct value was indicative of the presence of an increased number of copies of the gene and is implicit regarding the number of microorganisms [27]. For some of the analyzed samples, conclusive results were not obtained following the real-time PCR analysis, due to the presence of some inhibitory compounds in the DNA extracts (*B. edulis*, ascending segment; *P. ostreatus*, transverse and distal segments). With regard to the number of lactobacilli in the analyzed samples, there is a noticeable increase of *A. bisporus* consumption in all three segments of the large intestine. For all other samples, variations in the number of microbial types were determined depending on the sample and on the analyzed segments. As an example, in the case of *C. cibarius* consumption, a high number of lactobacilli were determined in the transverse section, whereas a small number of lactobacilli in the distal segment were determined following consumption of *B. edulis* and a small number of lactobacilli in the ascending colonic segment were also found following the consumption of *C. cibarius*.

### 3.4. Antioxidant Analysis

The total flavonoid content at the level of each colon segment is presented in Table 3. Because mushroom fungi are considered an important source of various bioactive compounds, the determination of the bioactive compound content in fungi is a prerequisite for a complete characterization of the biologic efficacy of mushrooms. The polyphenol and flavonoid content varied significantly, depending on the tested species of fungi. The consumption of *Boletus edulis* resulted in a maximum concentration of phenols in the transverse and descending segments of the colon, with an average of 46.09 mg gallic acid/g of ingested mushroom. Depending on the total amount of determined phenols, the order in phenolic concentration as a function of fungi type intake was as follows: *Agaricus bisporus > Cantharellus cibarius > Pleurotus ostreatus*. The increase in the total amount of phenols in the transverse section was ≤10% and in the descending section ≤20%. The lowering of phenols concentrations in the last segment was ≤5% for *B. edulis* and *A. bisporus*. It was observed that, both for phenols and flavonoids, the minimum determined amount was for the ascending segment of the colon. With regard to the flavonoids, the determined order varied according to the colon segment. A maximal peak was determined for *B. edulis* in the ascending and transverse segments (>100 mg quercetin/g ingested mushroom), and, for *P. ostreatus* (>125 mg quercetin/g ingested mushroom), a peak was determined in the descendant one. The increase in flavonoid concentrations with the consumption of *P. ostreatus* was approximately 46% and approximately 56% for *C. cibarius* consumption in the terminal segment of the colon.

**Figure 3:**
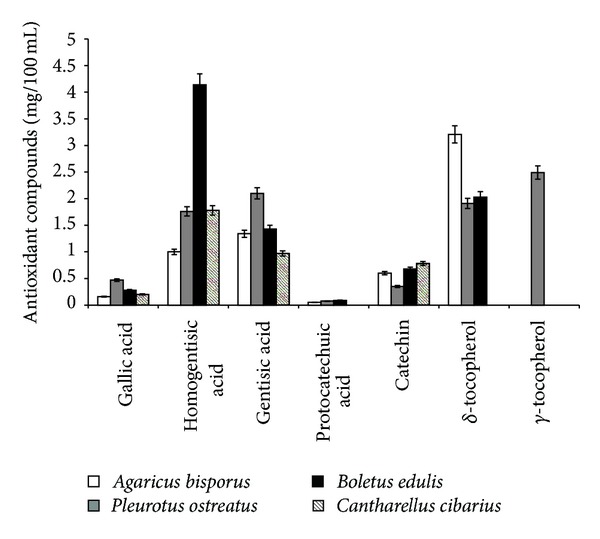
Average values of biomolecules with antioxidant efficacy.

During *in vitro* simulations, the analysis of various antioxidant biomolecules (phenolic acids, flavonoids, and tocopherols) induced by fermentative means following the consumption of the four types of mushrooms was also performed by HPLC (Figure 3). The gentisic acid was the highest determined amount, except for the consumption of *B. edulis *and* C. cibarius*, where the homogentisic acid was determined as having the highest released amount (4,143 and 1,786 mg/100 mL). Other identifiable phenolic compounds were gallic acid (0.161–0.476 mg/100 mL) and protocatechuic acid (0.051–0089 mg/100 mL), with the exception of *C. cibarius* consumption. Following the *in vitro* studies of flavonoids, only catechin was identified, with a maximum concentration determined for *C. cibarius* consumption. The maximum identified quantities corresponded to the distal section of the colon. 

Significant amounts of *δ*- and *γ*-tocopherol were also identified. *γ*-tocopherol was determined only with the consumption of *P. ostreatus* (2.49 mg/100 mL), and *δ*-tocopherol was not identified with *C. cibarius* mushroom consumption. The highest amount of *δ*-tocopherol was nevertheless identified for *A. bisporus* in the transverse colon.

Although polyphenols have been shown to degrade by the action of colonic microflora fermentation, the present study demonstrated that depending on the consumed mushroom type, the level of these molecules may differ. Determination of dry matter disappearance showed a reduced capacity for *A. bisporus*, of approximately 20%. For the other mushroom types, dry matter disappearance was within the limits of 30–40% in the transverse colon. In contrast, for *B. edulis*, the dry matter disappearance had a high value of greater than 70%. This determination was also supported by a complete digestion of the fungus observed in the final segment of transverse colon, as reflected also in a high level of polyphenols compared to other types of fungi. The fiber composition showed a different digestion capacity, which probably affects the polyphenol level in the colon segments. This ranged from 7 to 10%, which is inversely proportional to the values obtained for dry matter disappearance and to the amount of polyphenols present in the colon. Fermentation of the undigested part by colonic microflora is a means of preserving the colon health, having a more pronounced effect in the case of *B. edulis* consumption compared to all other mushroom types consumption. The fermentative capacity of this substrate is reflected in the products of metabolism, through a modulation of colonic microflora in the three colonic segments.

The fermentation of polysaccharides, fibers, and the biomolecules with an energetic role as well as the presence of vitamins in fruit bodies of different mushrooms caused an increase in the microbial microflora activity [28]. This process results in an increase in short-chain fatty acid synthesis that increases with the passing from one colonic segment to another. It was observed to be greater for *B. edulis* and *P. ostreatus* and less significant when *C. cibarius* is ingested, due to the antimicrobial effect on some types of microbial groups. A relatively constant reduced synthesis of both ammonia and lactate occurs following *A. bisporus* consumption.

The highest amount of acids (propionic acid) has not exceeded 30 *μ*mol/mL in the transverse colon segment. Compared with *B. edulis* and *P. ostreatus*, the other mushrooms had a mean decrease of approximately 10% for *C. cibarius* consumption and of approximately 23% for *A. bisporus*. The quantities of ammonia and lactate were lower in the ascending and transverse segments for *B. edulis* and *P. ostreatus* consumption, with the lowest increase in the descendent segment due to complete digestion of the mushrooms. The growth in the last segment did not exceed 5% (20 *μ*mol/mL). The effect on ammonia is similar to that of some prebiotics (inulin), which is beneficial for the normal functioning of the intestinal epithelial cells [29].

The biomolecules present in fungi, mainly the phenolic components, are known to play an important antioxidative role in their ability to inhibit free radicals. The correlation between the concentration of these types of biomolecules and the antioxidant activity is important in characterizing the biological action of a product. In this study, the *R* ^2^ coefficient, calculated for total phenolic content, correlated with the antioxidant activity, and the reducing power showed a linear relationship, which proportionally increased with the content of phenols [30]. The correlations between the antioxidant activity and the reducing power ranged from low to significant, ranging 0.7–0.911 (*P* < 0.0001) for the ascending segment, 0.34–0.722 (*P* < 0.0002) for the transverse segment, and 0.686–0.862 (*P* < 0.0005) for the descending segment. The most significant correlation was determined for *B. edulis* in all three segments. 

The correlation (*R* ^2^) between the total phenolic content and antioxidant activity was determined to be 0.55–0.995, *P* < 0.0004. The lowest value was calculated for *C. cibarius*, suggesting that the antioxidant activity was not determined exclusively by the phenolic component. Also in this case, as well as for flavonoids, the highest correlation was determined for *B. edulis*. The degree of correlation with the total amount of phenols/flavonoids increased with the transition from one segment to another of the colon, on average with approximately 30%. The power reduction however showed a correlation value (*R* ^2^) depending on the colon segments, that ranged 0.506–0.928 (*P* < 0.0003) for the ascending, 0.416–0.748 (*P* < 0.0004) for the transverse, and 0.22–0.833 (*P* < 0.0007) at the level of the descending segment. For *C. cibarius*, the correlation of the reduction power with various determined biomolecules was divided differently; if a correlation of 1.00 is considered the maximum value of *R* ^2^, the highest percentage (*≈*60%) is generated by the presence of the phenolic component. It was noted that, in the transverse segment, the decrease in *R* ^2^ value was correlated with a maximum fermentative capacity of the microflora and with a maximum digestion of ingested mushroom. The observation was based on the identification of a maximum amount of dietary fibers, which showed to be greatest for *C. cibarius* consumption and smallest for *A. bisporus*. This was also supported by an appropriate percentage of dry matter disappearance which correlated with the colon segment, with the quantity of each type of biomolecules, and with their release into the existing environment in GIS1 system. The high degree of correlations between these biochemical methods, for assessing the antioxidant capacity and the content in biomolecules with antioxidant effect, proved that these methods can be used to determine the effect of consumption of different types of mushrooms. Determining the correlation of the total amount of phenols, flavonoids, and carotenoids to consumed mushroom type in all three segments of the colon demonstrated the importance of validating the relationship between microflora and the levels of these biomolecules, as thus the antioxidant status.

A low to significant correlation was found between the number of *Lactobacillus* and *Bifidobacterium* strains from human colon and the levels of total phenols and antioxidant activity. For the ascending segment of the colon, the *R* ^2^ value between the amount of phenols and the number of lactobacilli (log CFU/mL) ranged 0.5637–0.815 (*P* < 0.0002), and 0.53–0.915 (*P* < 0.0005) for the transverse segment and 0.624–0.891 (*P* < 0.0003) for the descendent segment. Analysis of the results denoted a low correlation between various antioxidant compounds and the number of clostridia, coliforms, and staphylococci in different segments of the colon. The antioxidant capacity determined in the colon segments was also positively correlated with the number of lactobacilli (*R* ^2^ = 0.758), bifidobacteria (*R* ^2^ = 0.58), and enterococci (*R* ^2^ = 0.905) (*P* < 0.0005). These data demonstrated the major active role of phenolic components in antioxidant activities status in human colon and the dynamic relationship between the various groups of human microflora.

Mushroom consumption effect on antioxidant potential in the three regions of the human colon was determined using the *in vitro* simulation unicameral system GIS1. Upon entry into the human colon, the mushroom released some of the biomolecules in the stomach and small intestine. In the case of mushroom consumption, it was found that most biomolecules are released during the fermentative action of the microflora in the colon. This observation conflicts with other similar studies that have shown that most antioxidant biomolecules are released during gastric digestion, for example, when fruit such as apples are consumed [31].

A main contribution from this study is that mushroom consumption causes a gradual release of phenolic compounds from the beginning of digestion, which was confirmed by the determined antioxidant activity. This was experimentally established by the level of the determined phenolic compounds which is relatively constant, at least in the human colon regions. This may, in turn, explain in a novel way the significant role that mushroom consumption may play in preventing the negative effects of free radicals, with direct reference to antitumor protection. Besides these biomolecules, later when a digested fruit body reaches the colon, fibres are released, which, as demonstrated for *P. ostreatus* consumption, reduce the alterations induced by the tumor compounds [32]. Although generally there was a reduction of these colon tumor compounds with the transition through different compartments of the digestive tract [19], for mushrooms the effect is reversed due to their composition, being mainly disintegrated by a fermentative process and not by an enzymatic action or acid. 

Depending on the mushroom type consumed, there was a difference in the release of the types of antioxidant molecules studied. A differential action of the various antioxidant molecules within the colon environment was also noted and was dependent on the ingested mushroom type. A nonsignificant degradation of these molecules by the microflora in the colon was also noted to occur simultaneously. The amount released through the fermentation process was higher. If the respective quantities were decreased, this similar behaviour would occur as a result of the early release of acid and respective enzymatic digestion in the stomach and small intestine. According to the results obtained by using the GIS1, approximately 40% of total phenols and flavonoids present were released following acid and enzymatic digestion, and the rest was released in the colon by the fermentation process.

Overall, the real-time PCR analysis confirmed the microbiological analysis of different groups of microorganisms in simulated colonic microflora. These results denote a weak bifidogenic effect from ingestion of mushrooms, possibly due to their composition, known to have a strong antimicrobial effect. In the distal colon segment, the lowest concentration of bifidobacteria was observed as was already confirmed by previous studies on the level of these microorganisms following ingestion of prebiotics [29]. In addition, in a study on the *in vitro* simulation of human colonic microflora, it was noted that, contrary to inulin, the consumption of the four species of mushrooms stimulated the number of lactobacilli, with maximal effect depicted within the transverse segment of the colon. This effect was partially correlated with the total phenols released from the digestive process which is a direct indicator of colonic microflora action that caused an increase in antioxidant potential due to the process of digestion. All results represent new aspects concerning the effect that colonic bacteria and especially the anaerobic bacteria could have at this level. Nonpathogenic bacteria present a significant interest because of their antitumoral effects by the competition at the gut level and also because of their capacity to deliver different biologically active molecules. A superior selectivity over the cancer tissue is proved by anaerobic bacteria or facultative anaerobic bacteria attenuated strains [33].

The original aim of the research, the effects of the mushrooms on the colon microbiota, was demonstrated to be linked to the antioxidant activity, presence of different antioxidant molecules, amount of fiber, and fermentative capacity of microflora. Capacity of the colonic microflora to release the active molecules from mushrooms is a significant finding because it distinguishes between the effects of antioxidants versus fiber for bacterial growth. According to our research the amount of *Lactobacillus* and *Bifidobacterium* strains was correlated with both quantity of fiber and antioxidant molecules. The significance of the research results from the modulation capacity of human colon microflora by exogenous factors proved the consumption of food rich in active molecules with antioxidant properties. The results are valid only for these two wild edible mushroom species. The difference between mushrooms species is very high in terms of quantity of bioactive molecules and biologic activities.

Within a similar sample of porcini rosmarinic acid in the lyophilized extract was identified for the first time. However, following *in vitro* digestion this was not present. Thus, the absence of this compound may be due to the fermentative activity of microflora and to the digestion process which led to its degradation which started in the stomach and the small intestine [5]. Thus, the presence of rosmarinic acid would nonetheless increase the antioxidative value of mushroom consumption, as the antioxidant activity of the rosmarinic acid was shown to be higher than vitamin E depicted by its ability to protect cells from damage caused by free radicals and the reduction of various forms of cancer and atherosclerosis [34].

The results of the present study regarding the consumption of the four different species of mushrooms, using *in vitro* GIS1 simulator, showed a significant antioxidant activity in the human colon and an increase in the fermentative capacity of microflora. The synergy between the components with antioxidant effects and of fibers, which differed for each consumed fungi species, showed the possibility of improving the integrity of the colon. The study performed on the bioavailability of these compounds using GIS1 simulation system showed a high presence in the colon, leading to results that were comparable to those using extracts. Since results obtained by using GIS1 are similar to the results obtained from *in vivo* conditions, we can therefore understand the effect of the consumption of these various fungi species on the physiological functions of the human colon.

## Figures and Tables

**Table 1:** Average number of microorganisms (log CFU/mL) for the different microbial groups according to consumed mushrooms.

Bacterial group	Ascendant colon	Transversal colon	Descended colon
	Control		
Total anaerobes	7.32 ± 3.21	7.60 ± 1.85	7.20 ± 2.11
Facultative anaerobes	7.69 ± 0.71	7.78 ± 2.25	7.73 ± 2.34
Lactobacilli	7.08 ± 0.32	7.21 ± 0.76	7.23 ± 1.49
Bifidobacteria	6.87 ± 0.87	6.88 ± 0.23	6.97 ± 0.55
Enterococci	6.83 ± 1.01	6.45 ± 0.56	6.42 ± 1.03
Clostridia	6.67 ± 1.64	6.77 ± 1.21	6.84 ± 2.09
Coliforms	6.97 ± 1.65	7.03 ± 0.71	7.13 ± 1.07
Staphylococci	6.59 ± 0.79	6.51 ± 1.45	6.48 ± 1.35

	*Pleurotus ostreatus *		
Total anaerobes	7.10 ± 1.3	8.27 ± 0.76	8.21 ± 2.1
Facultative anaerobes	7.34 ± 1.1	7.00 ± 1.05	7.15 ± 0.11
Lactobacilli	7.25 ± 0.83	8.34 ± 1.58	8.55 ± 0.62
Bifidobacteria	5.32 ± 0.48	5.52 ± 2.49	5.47 ± 1.54
Enterococci	7.05 ± 0.39	7.63 ± 0.06	7.54 ± 0.13
Clostridia	7.01 ± 0.41	6.78 ± 0.25	6.85 ± 0.09
Coliforms	6.88 ± 0.17	7.48 ± 1.47	7.11 ± 1.59
Staphylococci	6.32 ± 0.43	6.47 ± 0.36	6.45 ± 0.52

	*Agaricus bisporus *		
Total anaerobes	7.64 ± 0.97	7.69 ± 8.10	7.97 ± 5.43
Facultative anaerobes	7.67 ± 4.40	7.88 ± 3.09	7.82 ± 5.42
Lactobacilli	8.09 ± 5.16	8.65 ± 3.79	7.74 ± 2.72
Bifidobacteria	5.91 ± 3.04	6.11 ± 1.07	6.17 ± 0.63
Enterococci	7.81 ± 1.23	7.85 ± 0.64	7.76 ± 4.49
Clostridia	7.80 ± 6.17	6.78 ± 3.90	6.84 ± 1.53
Coliforms	7.99 ± 2.33	7.65 ± 8.01	7.17 ± 9.75
Staphylococci	6.78 ± 0.76	6.94 ± 1.13	6.98 ± 3.21

	*Boletus edulis *		
Total anaerobes	8.5 ± 0.86	8.32 ± 1.13	8.72 ± 3.52
Facultative anaerobes	8.43 ± 1.07	8.40 ± 2.16	8.78 ± 0.38
Lactobacilli	7.60 ± 0.73	7.83 ± 0.83	6.69 ± 2.98
Bifidobacteria	5.13 ± 4.07	5.59 ± 0.79	5.45 ± 1.01
Enterococci	8.15 ± 0.23	8.07 ± 0.65	8.43 ± 1.63
Clostridia	7.19 ± 0.17	6.94 ± 0.19	7.26 ± 0.07
Coliforms	6.62 ± 0.37	6.49 ± 0.06	6.61 ± 0.34
Staphylococci	6.56 ± 0.83	6.51 ± 0.23	6.52 ± 1.17

	*Cantharellus cibarius *		
Total anaerobes	8.75 ± 2.44	7.98 ± 1.43	8.02 ± 0.67
Facultative anaerobes	8.06 ± 4.89	8.20 ± 0.71	8.32 ± 0.35
Lactobacilli	7.44 ± 4.17	8.49 ± 0.17	6.02 ± 0.19
Bifidobacteria	5.44 ± 3.23	4.85 ± 1.32	4.25 ± 0.45
Enterococci	7.31 ± 2.69	7.66 ± 3.27	7.82 ± 0.11
Clostridia	6.35 ± 1.62	6.22 ± 2.75	6.00 ± 0.53
Coliforms	6.41 ± 4.22	6.71 ± 2.65	6.90 ± 0.03
Staphylococci	6.78 ± 3.98	6.04 ± 3.73	5.51 ± 0.59

**Table 2:** Average Ct values (threshold cycle) of samples in the various colon segments following mushroom consumption.

Sample	Ct values	
	General bacteria	Lactobacilli
Ascending colon		
* Pleurotus ostreatus *	—	20.39 ± 0.009
* Agaricus bisporus *	13.04 ± 0.006	17.66 ± 0.003
* Boletus edulis *	—	—
* Cantharellus cibarius *	—	28.72 ± 0.007
Transverse colon		
* Pleurotus ostreatus *	—	—
* Agaricus bisporus *	15.36 ± 0.00	20.73 ± 0.003
* Boletus edulis *	17.47 ± 0.008	25.95 ± 0.001
* Cantharellus cibarius *	13.97 ± 0.008	18.15 ± 0.001
Descending colon		
* Pleurotus ostreatus *	—	—
* Agaricus bisporus *	10.96 ± 0.003	16.53 ± 0.005
* Boletus edulis *	—	28.95 ± 0.00
* Cantharellus cibarius *	—	18.88 ± 0.007

**Table 3:** Total phenols and flavonoid content in the various colon segments following mushroom consumption.

Content	Ascendant colon	Transversal colon	Descended colon
	*Pleurotus ostreatus *		
Total phenols	19.5 ± 0.21	21.4 ± 1.31	24.47 ± 1.56
Flavonoids	97.5 ± 0.43	67.86 ± 3.27	125.36 ± 5.65

	*Agaricus bisporus *		
Total phenols	21.2 ± 3.29	30.14 ± 0.05	29.32 ± 0.31
Flavonoids	50.51 ± 2.61	56.85 ± 2.93	60.75 ± 4.72

	*Boletus edulis *		
Total phenols	40.22 ± 1.91	46.53 ± 2.74	45.65 ± 0.48
Flavonoids	103.87 ± 0.53	95.43 ± 1.75	83.6 ± 0.58

	*Cantharellus cibarius *		
Total phenols	19.71 ± 4.57	21.07 ± 0.14	25.85 ± 1.42
Flavonoids	18.26 ± 2.95	20.11 ± 0.56	45.42 ± 0.85
